# A Technology Training Program to Alleviate Social Isolation and Loneliness Among Homebound Older Adults: A Community Case Study

**DOI:** 10.3389/fpubh.2021.750609

**Published:** 2021-11-18

**Authors:** Frances N. Jiménez, Joan F. Brazier, Natalie M. Davoodi, L. Carter Florence, Kali S. Thomas, Emily A. Gadbois

**Affiliations:** ^1^Department of Health Services, Policy and Practice, Brown University School of Public Health, Providence, RI, United States; ^2^Center for Gerontology and Healthcare Research, Brown University School of Public Health, Providence, RI, United States; ^3^Meals on Wheels America, Arlington, VA, United States

**Keywords:** information and communication (ICT), community-based organizations (CBOs), older adults, social isolation, loneliness

## Abstract

Despite substantial evidence of the negative health consequences of social isolation and loneliness and the outsized impact on older adults, evidence on which interventions are most effective in alleviating social isolation and loneliness is inconclusive. Further complicating the translation of evidence into practice is the lack of studies assessing implementation and scalability considerations for socialization programs delivered by community-based organizations (CBOs). Our primary objective was to describe the implementation barriers, facilitators, and lessons learned from an information and communication technology (ICT) training program aimed at reducing social isolation and loneliness for homebound older adults in a home-delivered meals program. Participants received in-home, one-on-one ICT training lessons delivered by volunteers over a 14-week period with the goal of increasing social technology use. To assess implementation facilitators and barriers, 23 interviews were conducted with program staff (*n* = 2), volunteers (*n* = 3), and participants (*n* = 18). Transcripts were analyzed using thematic analysis. Aspects that facilitated implementation included the organization's existing relationship with clientele, an established infrastructure to deliver community-based interventions, alignment of intervention goals with broader organizational aims, and funding to support dedicated program staff. Challenges to implementation included significant program staff time and resources, coordinating data sharing efforts across multiple project partners, participant and volunteer recruitment, and interruptions due to COVID-19. Implications of these facilitators and barriers for scalability of community-based ICT training interventions for older adults are described. Lessons learned include identifying successful participant and volunteer recruitment strategies based on organizational capacity and existing recruitment avenues; using a targeted approach to identify potential participants; incorporating flexibility into intervention design when working with the homebound older adult population; and monitoring the participant-volunteer relationship through volunteer-completed reports to mitigate issues. Findings from this formative evaluation provide insight on strategies CBOs can employ to overcome challenges associated with implementing technology training programs to reduce social isolation and loneliness for older adults, and thus improve overall well-being for homebound older adults. Recommendations can be integrated into program design to facilitate implementation of ICT programs in the community setting.

## Introduction

Social isolation and loneliness are significant threats to physical and mental health, particularly among older adults. Both are associated with poor health outcomes including comorbid conditions (1), cognitive decline (2, 3), and mortality (4). Homebound older adults, comprising 8.3% of community-dwelling older adults in the United States (5), are especially at risk of social isolation and loneliness due to mobility limitations caused by chronic illness, cognitive decline, or injury (6, 7). In fact, being homebound and socially isolated have a synergistic effect on increasing risk of mortality (8).

Meals on Wheels America (MOWA), the leadership organization that supports the national network of Meals on Wheels (MOW) programs, aims to alleviate social isolation and loneliness among homebound MOW clients. Studies suggest that receiving home-delivered meals through MOW can reduce loneliness and improve psychological well-being among homebound older adults due to the social interaction that accompanies meal delivery (9–11). However, some homebound clients need more social connection than provided at the point of delivery, prompting MOWA to expand programming to focus specifically on social connection. Formal efforts currently delivered through MOW programs to address isolation and loneliness lack strong evaluation and have not been scaled widely (12). To bridge this gap, researchers worked with MOWA and a MOW program in Rhode Island to pilot an intervention aimed at alleviating social isolation and loneliness through technology. The program, called Talking Tech, addresses barriers to technology adoption in homebound older adults by providing in-home, one-on-one training to promote digital literacy, virtual connection with family and friends, and participation in a virtual senior center.

### Background

In response to physical distancing orders enacted to mitigate the spread of coronavirus 2019 (COVID-19), organizations and researchers alike seek to develop and deliver solutions to combat social isolation and loneliness among older adults, who are at outsized risk of complications caused by COVID-19 (13, 14). Even before the pandemic, researchers, policymakers, healthcare professionals, and social service providers noted social isolation and loneliness as priorities to address for older adult health (15, 16), yet limited evidence-based options exist for socially isolated or lonely homebound older adults (17–19).

Information and communication technology (ICT) interventions are one potential solution for addressing social isolation and loneliness among older adults (20, 21) by helping them connect to a larger community, gain social support, engage in activities of interest, and boost self-confidence (18, 20). Individualized ICT training has been shown to increase older adults' technology adoption and acceptance (22). ICT interventions can aid homebound, isolated older adults in socialization by allowing them to engage with others from within their home (18, 19). However, barriers, such as lack of technology knowledge, support, broadband availability, and cost, limit the adoption of ICT among older adults (23, 24). These challenges are exacerbated among homebound persons, many of whom are low-income and lack access to technology training and support (25). If tailored to meet the needs of homebound older adults, ICT interventions could fill the gap in needed social isolation and loneliness programs for this population.

The COVID-19 pandemic underscores the need for community-academic partnerships to translate evidence on social isolation and loneliness interventions into practice among frontline service providers, as well as cross-sector collaborations to leverage existing resources and infrastructure to enable continued delivery of services to older adults (13, 26). In the social isolation and loneliness intervention literature, both assessment of community-oriented implementation processes and scalability and sustainability considerations for community-based organizations (CBOs) are not adequately addressed, limiting the ability of CBOs to apply findings to their own work. To encourage adoption of research-informed socialization programming for homebound older adults by CBOs, this community case study describes organization-level facilitators, barriers, and lessons learned from an ICT training program developed *via* a community-academic partnership.

## Context

**Figure 1:**
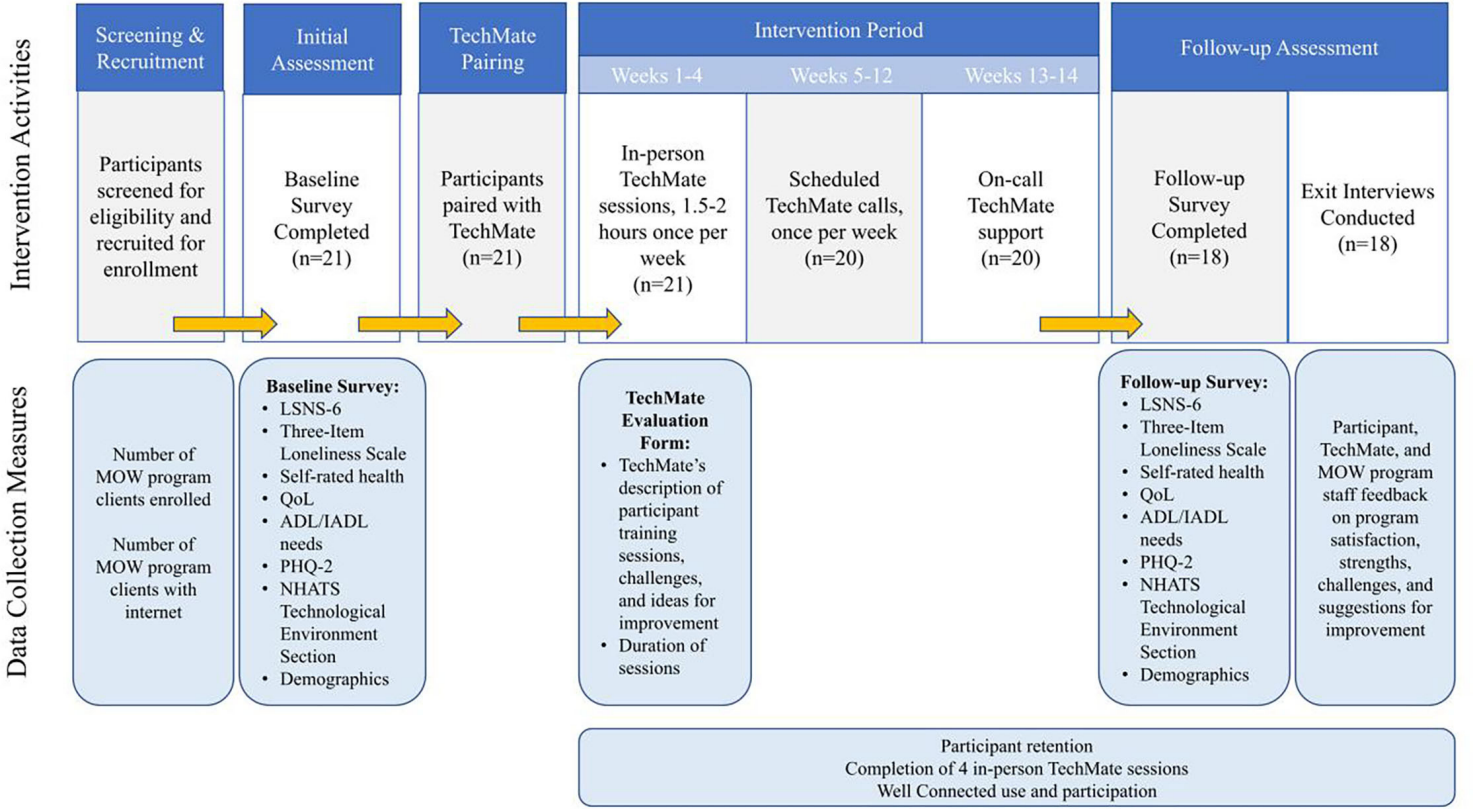
Talking Tech intervention activities and timeline.

Talking Tech is a 14-week in-home, one-on-one, volunteer-delivered ICT training intervention. Older adults were paired with a volunteer, called a TechMate, and provided a Surface Pro tablet and internet connection, if needed, to learn how to use a computer device and the internet to socially connect with new and existing contacts *via* 1.5–2 h prepared modules. Talking Tech introduced participants to Well Connected, a national phone and internet-based virtual community that offers over 70 weekly activities, classes, and support and conversation groups. Program activities and timeline are described in Figure 1. With support from MOWA, Talking Tech was implemented at Meals on Wheels Rhode Island (MOWRI) from October 2019 to May 2020. Talking Tech is a collaboration between multiple organizations, including: Tech4Life, a technology training company; Covia, which operates Well Connected; MOWA; MOWRI; and a research university (Table 1). The study team's university IRB evaluated the study and determined it not to be human subjects research.

**Table 1:** Intervention partner roles and responsibilities.

**Partner organization**	**Primary role/responsibilities**
Meals on Wheels America	Funded and managed Talking Tech implementation, provided ongoing support for MOWRI including training
Meals on Wheels Rhode Island	Coordinated Talking Tech implementation and delivery, including recruiting volunteers and participants, hosting TechMate training sessions, and providing support to participants
Tech4Life	Developed TechMate training session material and module lesson plans, conducted TechMate training
Covia	Operated Well Connected phone and computer sessions; monitored and shared Talking Tech participation in Well Connected sessions
Brown University School of Public Health	Provided evaluation and research support, including designing and conducting baseline and follow-up survey questionnaire and exit interview protocols, and analyzing data

Talking Tech was designed by MOWA and the university team, which met weekly to determine program milestones and deliverables. Feedback was sought from MOWRI leadership. During implementation, all partner organizations met weekly to discuss successes and challenges and further refine program delivery. For example, partners discussed improvements to TechMate training in light of TechMate questions and challenges, including additional training on hotspot set up and device troubleshooting and parameters around escalating participant concerns.

## Essential Elements of the Intervention

MOWRI recruited Talking Tech participants from their home-delivered meal program, using multiple methods including meal delivery driver referrals, program fliers accompanying delivered meals, and email outreach to clients who self-identified as lonely in an annual client survey. Participants were 60 years or older and homebound. Twenty-one MOWRI clients enrolled in Talking Tech. Volunteers were initially recruited from the existing pool of MOWRI's ~700 annual volunteers through a volunteer appreciation event, by email, and by social media. Due to challenges recruiting existing MOWRI volunteers described below, MOWRI then solicited volunteers through an online volunteer portal, corporate partnerships, and posting fliers at a local university. TechMates were required to have existing computer and internet knowledge. Eighteen individuals volunteered as TechMates.

Tech4Life trained volunteers in a 1-h training session. Volunteers were given a manual containing program goals, expectations for TechMates and participants, module lesson plans with step-by-step instructions and objectives, participant worksheets, and a Well Connected catalog with class schedules. Instructional handouts on setting up the tablet and appropriate shortcuts, setting up a hotspot internet connection, and accessing Well Connected were also included. Training was offered in-person on two occasions in September and November 2019 and by video recording. Thirteen volunteers attended the in-person training and 7 volunteers viewed the recorded training; upon completing training, two individuals withdrew from participating as TechMates due to self-described limited technology comfort and knowledge.

To understand participant experiences and satisfaction with Talking Tech, researchers conducted semi-structured interviews with 18 of the 21 participants after intervention completion; one participant withdrew and two participants were unable to be reached. Interview questions focused on technology usability, perceived impact of internet and Well Connected use on well-being, and satisfaction with Talking Tech components. Three TechMates and two MOWRI program staff were interviewed to understand implementation experiences, facilitators and barriers to program implementation and delivery, observed participant satisfaction, and suggestions for improvement. Initially, participant interviews were conducted within 2 weeks of intervention completion in participants' homes with written informed consent; however, due to COVID-19, only four interviews were conducted in-person. Remaining interviews were conducted by telephone and verbal consent was obtained. Participant interviews lasted between 20 and 90 min. Interviews with staff and TechMates were conducted by telephone after the intervention period and verbal informed consent was obtained. TechMate interviews lasted 40–60 min and staff interviews were 15–60 min. All interviews were audio recorded with consent and transcribed.

During the first 4 weeks, TechMates submitted a report to the Talking Tech coordinator at MOWRI after each participant interaction, documenting the duration of the interaction, participants' questions, participant comfort level and interest with the technology, including the tablet, internet, and Well Connected, challenges, and ideas for program improvement (Supplementary File 1). While outside of the scope of this paper, which presents findings on facilitators, barriers, and lessons learned from intervention implementation, additional quantitative data were collected to evaluate outcomes of the intervention. A resulting manuscript is in progress.

### Analysis

Qualitative data were analyzed using a thematic analysis approach (27). Three researchers coded the first three participant interviews independently and met to develop a preliminary coding scheme. The coding scheme was then revised after four researchers independently analyzed and discussed all 23 interviews. Transcripts were double coded in rotating pairs to ensure consistency. TechMate reports were reviewed by one researcher for content relevant to lessons learned regarding program implementation. We recorded coding definitions, decisions, and ideas about emerging themes in an audit trail to ensure analytic rigor (28). Qualitative data from interviews and TechMate reports were analyzed using NVivo Version 12 Plus^1^.

## Results

In this paper, we describe facilitators, barriers, and lessons learned of Talking Tech implementation from the MOWRI perspective, as identified from interviews with Talking Tech participants, volunteers, and MOWRI program staff (Table 2).

**Table 2:** Themes of Talking Tech implementation facilitators, barriers, and lessons learned.

**Theme/Subtheme**
**Facilitators**
An existing relationship with and history of serving the target population allowed for identification of clients at-risk of being socially isolated or lonely and facilitated trust among participants.
Alignment of ICT program aims with organizational mission and existing infrastructure garnered support from organization leadership.
Funding supported a dedicated part-time staff member to coordinate program implementation and allowed for purchase of program materials.
Subtheme: A part-time program coordinator was critical to successful ICT program implementation and operation.
**Barriers**
Program staff time and organizational resources needed to implement the program were greater than anticipated.
Data sharing among project partners was inhibited by system and/or organization privacy requirements and sharing restrictions.
Volunteer and participant recruitment were the most challenging component of program implementation.
Subtheme: Potential volunteers and clients were hesitant to join Talking Tech due to the time commitment.
Physical distancing orders from the COVID-19 pandemic led to interruptions in in-person TechMate sessions.
Subtheme: Most participants preferred to delay lessons and resume in-person sessions once safe to do so rather than transition to telephone sessions.
**Lessons learned**
Identify successful participant and volunteer recruitment strategies based on organizational capacity and existing recruitment avenues.
Use a targeted approach to identify potential participants who are socially isolated or lonely.
Subtheme: Participants and volunteers who were most successful with completing the ICT program had some prior technology experience, suggesting the need for screening questions on technology experience during recruitment.
Provide program flexibility when working with the homebound older adult population.
Subtheme: ICT training programs may not be suitable for all older adults, depending on interest and pre-existing technology knowledge.
Subtheme: Volunteers, participants, and MOWRI staff expressed the need for on-call expertise to assist with troubleshooting complex technology issues.
Subtheme: Adequate volunteer training on working with older adults and technology is necessary.
Implement a process for ongoing, remote monitoring of the participant-volunteer relationship, such as weekly reports, in order to intervene and resolve participant-volunteer issues, when appropriate.

### Implementation Facilitators

Aspects that facilitated Talking Tech implementation included the existing relationship between MOWRI and its clientele; an established infrastructure to deliver community-based programs; alignment of intervention goals with broader organizational aims; and funding to support dedicated program staff and materials.

Both participants and organization staff noted the importance of participants' existing relationship with the delivery organization. For MOWRI, it enabled identification of potentially socially isolated homebound older adults, allowing for a targeted approach to participant recruitment. As existing clients, intervention participants had already developed a relationship with MOWRI personnel. In some instances, participants' regular meal delivery drivers also served as their TechMate. One participant, speaking to the relationship they had developed with their TechMate who also volunteered as their meal delivery driver stated*, “[TECHMATE NAME] delivers my meals so I had met him and knew him but he's just, it was nice having him here… I was very comfortable”* (Participant 1). The existing relationship with MOWRI facilitated trust among participants and familiarity with whom to call if questions or issues arose. One participant described turning to the Talking Tech coordinator at MOWRI when they encountered technology issues since they already knew and were comfortable calling the organization phone number: “*I tried to use [the tablet] quite a few times, and then I got stuck. I don't know what I did, but I called [Program Coordinator]. and she was able to get me out”* (Participant 8).

The home-based, volunteer-delivered model used to deliver Talking Tech was similar to the design of the home-delivered meal program in which volunteers deliver meals to clients homes, which allowed MOWRI to utilize existing volunteer recruitment and training workflows in the operation of Talking Tech. Speaking to the suitability of Talking Tech with existing workflows, one MOWRI staff member noted, “*I definitely think it's something that could be implemented here, for sure. It's a good fit”* (MOWRI Staff 2). Additionally, MOWRI leadership stated that Talking Tech's aims to reduce social isolation and loneliness fit “*perfectly*” within their organization's mission, as well as their push to modernize services (MOWRI Staff 1). These factors led to leadership supporting the program, which facilitated prioritization of meeting program goals among staff.

Funding to support the intervention was noted as a critical resource, as it allowed for dedicated staff time and the purchase of necessary materials (e.g., hotspot internet devices, tablets). Staff reported that low digital literacy was common among their older adult clientele, which contributed to significant time spent recruiting participants, fielding questions, and supporting participants. Staff reported that to properly support MOW clients, at least one part-time staff member was needed to coordinate program implementation and delivery. One MOWRI staff member described the Talking Tech coordinator as key to the success of the implementation of the intervention, stating, “*Once we moved [her] onto the project, I felt like we really kind of were able to better deliver on it. So, that was having a staff member that had organization and communication and management skills.”* In addition to enabling MOWRI to dedicate a part-time staff member, funding ensured participant access to tablets and hotspot internet connections. By providing tablets and internet free of cost to participants, the intervention was accessible to the low-income, homebound older adult population that the organization serves. Program leadership noted that Talking Tech “*created an opportunity for [clients] to get connected by breaking down barriers, such as access to a computer or access to the internet”* (MOWRI Staff 1), and that the tablet was the “*biggest benefit*” because “*clients that participated and received the Surface Pro in all likelihood never would've been able to purchase such a thing on their own*” (MOWRI Staff 2).

### Implementation Barriers

Challenges to implementation included the time- and resource-intensity of the intervention for MOWRI; data sharing restrictions across partners; participant and volunteer recruitment; and interruptions in program delivery due to the COVID-19 pandemic.

Organization staff reported that they had not anticipated how much time and effort would be required for implementation. MOWRI leadership stated:

“*It was a very labor-intensive project. Coordinating the volunteers, the seniors, so on and so forth, that we would have been able to maybe do a little bit more if we had a full-time person really focused on the work” (MOWRI Staff 1)*.

The Talking Tech coordinator described the program as “*much more time involved*” and “*a lot more daily work*” than they had anticipated due to “*all of the calls*” (MOWRI Staff 2). Recruitment calls were overwhelming not only because of the “*volume of calls*” but also because each conversation with a potential participant “*was a very long conversation*” due to the “*isolated and lonely*” nature of clients (MOWRI Staff 2). In addition to answering client and Talking Tech participant questions about the program, the Talking Tech coordinator reported that much of the unanticipated lift of the program involved acting as additional technology support and functioning as a liaison between participants and volunteers. Additionally, the coordinator reported spending unanticipated time outside business hours resolving logistical issues arising from tablet set up and hotspot issues.

While collaboration between partner organizations was necessary to develop and deliver the program without requiring greater resources from MOWRI, issues with implementing data sharing processes led to data tracking and reporting issues that impacted efforts to assess participation in Well Connected and TechMate lessons. Changes in data privacy policies prevented Covia from sharing complete records of participants' Well Connected use. To remedy this situation, Covia asked TechMates to track participant use of Well Connected and tell participants to include the tag “RI” in their username to identify participation in Talking Tech. Inconsistency among TechMates in submitting weekly TechMate reports complicated MOWRI and the university study team's ability to track TechMate lesson completion. The Talking Tech coordinator described “*some confusion”* among TechMates regarding when TechMate reports should be submitted, and that while she was able to “*chase down”* some reports she did not have the time to collect all reports (MOWRI Staff 2). The university team then tracked completion *via* participant interviews for evaluation purposes.

MOWRI staff described participant and volunteer recruitment as the “*greatest challenges*” encountered during implementation and that they “*did not anticipate that we would have to work so hard at it*” (MOWRI Staff 2). While MOWRI had pre-existing clientele and volunteer pools, the organization struggled initially to identify clients who were interested in participating in a technology-based program. The Talking Tech coordinator noted that clients declined participating because they “*felt that they were just too old to learn something new,”* were “*hesitant to have a stranger in their home,”* or that “*they didn't want to make a commitment of meeting with somebody once a week”* because it was seen by clients as “*an extra responsibility*,” despite its design to be a benefit. Additionally, MOWRI exhausted many channels to recruit volunteers, including both within and outside of the organization. Staff reported difficulty “*getting enough people to sign up*” as volunteers (MOWRI Staff 2), despite having a large volunteer pool, in part due to the multi-week time commitment required of TechMates.

Due to the COVID-19 pandemic, many participants' training lessons were interrupted, and some stopped altogether. While MOWRI encouraged TechMates to continue lessons *via* telephone, many participants expressed disinterest in continuing lessons remotely during the pandemic. They preferred to either wait until the risk of disease transmission dissipated and social distancing restrictions were lifted, or they turned to family members for support.

“*[W]hen the pandemic struck, we had to cease all in-person visits. Every TechMate was encouraged to continue the program via telephone. […] For some clients, I don't know if they felt like they couldn't do it over the phone because that was just too difficult. They had a hard enough time understanding things in person” (MOWRI Staff 2)*.

However, MOWRI staff noted:

“*We discovered that [Talking Tech] was even more needed. During COVID-19, when Meals on Wheels recipients really didn't have any access to family members or friends, and we saw many more seniors at that point trying to access online resources” (MOWRI Staff 1)*.

### Lessons Learned

Interviews yielded a wealth of information regarding lessons learned. These included the importance of identifying successful participant and volunteer recruitment strategies to inform future recruitment efforts; using a targeted approach to identify potential participants and volunteers; incorporating flexibility into intervention design when working with the homebound older adult population; and monitoring the participant-volunteer relationship through TechMate reports to mitigate issues.

Findings suggest that a targeted approach to identifying appropriate participants and volunteers needs to be considered when designing a technology-based program. As a pilot intervention, the purpose of this study was to determine which processes, including recruitment methods, should be implemented in a larger intervention. MOWRI staff identified targeting clients with an email address and who self-reported as lonely was the most effective participant recruitment strategy. However, the study team found that some participants were hesitant to discuss experiences of social isolation or loneliness with the research team during surveys and interviews, highlighting potential challenges for targeting and engaging isolated or lonely older adults in this work. The Talking Tech program coordinator at MOWRI described the easiest clients to recruit as those “*who had already indicated that they were comfortable using the internet… and who were open to an additional opportunity for something that would make them feel connected to the outside world”* (MOWRI Staff 2). While challenges in recruitment were in part due to client hesitation, those who ultimately participated found free access to a computer device and online programming a strong incentive to join:

“*The people who decided to participate and saw it as a very positive thing were floored that there was a program that was going to give them [a tablet]. They were very interested about the kinds of programming that they would have access to” (MOWRI Staff 2)*.

Given the range of technology comfort and experience among TechMates, the Talking Tech coordinator suggested screening potential TechMates based on technology knowledge and skills, stating “*a volunteer pre-survey to give folks to judge their level of knowledge and comfort with technology would be good, especially considering that we looked to our existing volunteer base, which is largely older folks themselves”* (MOWRI Staff 2). Volunteers, participants, and MOWRI staff also supported the recommendation of having a dedicated on-call technology support personnel to consult for advanced technology issues. Such a resource would expand the technological expertise available to participants and reduce the amount of time the Talking Tech coordinator spent fielding participants' technology-related questions. One TechMate suggested, “*I don't know if there's a way to have a tech person who's assigned to this study that can help the participants. …That would be really great”* (TechMate 2). As mentioned previously, two potential volunteers withdrew from participating after attending a training session because they felt that they would be unable to perform the technological tasks required of a TechMate, suggesting the need for targeted recruitment strategies and TechMate training evaluation to assess the effectiveness of training in preparing volunteers. While TechMates were asked about the effectiveness of training in interviews, no formal evaluation of TechMate training was performed in this pilot study. TechMates who were interviewed reported that the training was sufficient, however all three TechMates had extensive prior computer and internet experience.

Further, organizations should consider the aims of an ICT training program and who the corresponding target audience is in order to tailor recruitment efforts. The program coordinator noted that while a broader program goal may allow a wider reach, it may be more difficult to implement and evaluate compared to a goal with a specific audience in mind. While the specific goal of Talking Tech was to reduce social isolation and loneliness with a broader aim of increasing access to and use of technology, MOWRI staff stated that:

“*The [participants] that felt most comfortable using the technology had used some form of the internet or technology before. The folks that were targeted for this, the most elderly folks who did sign up and who had never used the internet on their own or anything were the folks that really became easily frustrated” (MOWRI Staff 2)*.

At the same time, the Talking Tech coordinator described needing to be flexible in the approach to working with homebound older adults. As many homebound organization clients have multiple chronic conditions that cause health complications, the coordinator reported participants canceling lessons with volunteers at the last minute due to health-related problems as a common challenge to carrying out training lessons. When working with the homebound older adult population, organizations should be cognizant of the additional barriers they face to participating in a weekly program and build flexibility into programs to accommodate participant needs. As the coordinator stated, “*[clients'] top concerns were being able to utilize [the tablet] and the difficulty associated with accessing the [Well Connected] programs*,” (MOWRI Staff 2), and as such volunteers should be prepared to address participants' technology concerns. TechMates described addressing clients' vision and dexterity impairments by changing font sizes and screen contrast and supplying stylus pens or computer mouses for those who were unable to use the tablet trackpad or touchscreen.

In addition to flexibility and accommodating needs, TechMates who were not prior MOWRI meal delivery drivers noted that training on communicating with older adults and making accommodations for participants with audio, visual, or mobility impairments would be helpful additions to volunteer training. One TechMate spoke to this need, saying:

“*I had worked with older adults before [.] so I felt fairly comfortable working with that population. But maybe others who haven't worked with older adults much would benefit from some guidance. And then maybe going into someone else's home, how you kind of navigate that setting” (TechMate 3)*.

While three TechMates were existing MOWRI volunteers who had received prior training and were accustomed to working with older adults in an individualized manner, these recommendations suggest that such interpersonal training is imperative for programs involving volunteers who work with older adults. For organizations with existing volunteer-based programs, volunteer-delivered ICT training programs can capitalize on or supplement existing volunteer training sessions.

Finally, MOWRI monitored the participant-volunteer relationships over the course of the intervention *via* weekly volunteer-submitted TechMate reports. These reports allowed MOWRI to identify emerging issues and intervene before escalation. For example, staff noticed *via* TechMate reports that one participant was becoming increasingly disengaged from learning as the intervention progressed, with the TechMate noting, “*She shows less interest as she considers it too confusing for her”* after the second lesson. The program coordinator was able to contact the participant, who ultimately decided to withdraw from the program, and reassign the TechMate volunteer to a new participant. The TechMate, who was highly engaged in Talking Tech, reported that this reassignment led to a rewarding relationship with their new participant and the participant's successful engagement with the program. In TechMate reports, the TechMate described the participant as “*excited about learning”* and that “*she responded well to instruction and is excited to do her ‘homework' as she calls practicing basic use of the computer.”*

## Discussion

This community case study reveals facilitators and barriers of implementing and delivering a community-based ICT training program for homebound older adults. We also include lessons learned and considerations for scalability and sustainability that may aid other CBOs in developing and implementing socialization programs for homebound older adults.

While social isolation and loneliness worsened during the COVID-19 pandemic (29), one study shows that frequent internet use during the pandemic buffered older adults against depression and declines in quality of life (30), suggesting that ICT training programs can address social isolation and loneliness in older adults both during and beyond the pandemic. The versatility of technology is a significant benefit of these interventions, as they have great potential for tailoring to individuals' specific needs. Prior research, as well as our own results, indicate that flexibility in program delivery is a necessary characteristic for successful technology training programs, as they allow for goal-motivated learning and individualization to accommodate participants' needs and abilities (31). To ensure flexibility and improve suitability and acceptance, programs can incorporate the end user throughout program design and development (32, 33).

To be effective among older adults, technology-based interventions must also incorporate elements of supportive training and ongoing assistance to overcome barriers specific to older adults, such as lack of experience, technology illiteracy, and fear of using technology (21, 34). As our findings demonstrated, the impact of the COVID-19 pandemic on the ability to conduct in-person lessons highlights the importance of in-person technology training for older adults with limited digital literacy. In another technology training intervention, internet and device training and support improved older adults' confidence and competence in technology use (34). The success of ICT interventions with older adults is also dependent on high-quality communication. When online interactions are perceived to not be reciprocal or rewarding, technology use can lead to increased social isolation in older adults (18). CBOs wanting to implement ICT training programs should recognize that while many interventions have been effective in reducing social isolation, the technology itself does not alleviate social isolation.

Our findings highlight recruitment and adherence considerations. During the program design phase, organizations should consider the interests, needs, and experiences of the target population in concert with the resources and approaches available to deploy for recruitment. We found that a targeted recruitment strategy involving direct outreach to self-identified lonely clients with an email address was the most successful recruitment approach. In order to perform targeted recruitment, programs will need systems in place to capture participant characteristics of interest, such as internet or technology use or comfort, assessed here by having an email address, and possible loneliness or social isolation. To identify who may be the most likely to benefit from the intervention, CBOs should consider using a validated screening scale such as the Upstream Social Isolation Risk Screener (U-SIRS) (13) to assess social isolation in clients. The varying interest and success among MOWRI clients also suggest that an ICT training program such as the one described here may be most suitable for homebound older adults with prior experience using technology or a strong interest in developing these skills. Organizations should anticipate varying levels of willingness to learn how to use technology and comfort with technology among older adults [(35); Brazier et al., 2021, unpublished manuscript]. To improve older adults' willingness to adopt technology, CBOs can develop strategies to address factors known to influence adoption, including privacy concerns, perceived value of technology, perceived impact on quality of life, and confidence in learning a new skill (36). Additionally, disparities in access to technology among older adults should be considered, as inequities could influence receptiveness to and comfort with technology. The so-called digital divide, where access to technology is limited among older adults who are BIPOC, low-income, or reside in rural areas (25, 37), must be considered by organizations seeking to implement these services.

Considerations central to scaling and sustaining ICT training interventions include the cost and resources needed to deliver such a program, including physical materials, staff, and training. Talking Tech's use of a volunteer-delivered model can be scaled with program expansion as layperson-delivered interventions can be brought to scale more quickly than interventions requiring licensed professionals. CBOs must consider recruitment strategies and balance the demand for volunteers and the available resources for a 14-week intervention such as Talking Tech. The experience of MOWRI suggests that a part-time or full-time staff position is necessary to coordinate implementation and delivery of the program, as participants will likely reach out to the organization directly with questions or concerns. Given the issues and concerns faced by the Talking Tech coordinator, we recommend that an individual with a background in social work is best suited to coordinate an ICT training program.

Difficulties with tracking enrollment and Well Connected participation highlight the complexity of implementing an intervention involving multiple collaborating organizations. Data collection and monitoring challenges are common in health-related cross-sector collaborations, and overcoming these challenges are integral to success (38). In our experience implementing Talking Tech, navigating multiple data collection systems inhibited data sharing and monitoring between partners. In order to build the evidence base for effective social isolation and loneliness interventions and support evaluation efforts, securing financial support and expertise for integrated data collection and monitoring processes will be critical.

### Limitations

One limitation of this pilot study is the small sample size of homebound older adults. However, as a pilot study reporting qualitative data, findings are not intended to be generalizable to other populations and are meant to be used to refine program development for future expansion. Additionally, interviews were conducted with only three volunteers and therefore may not capture the diversity of experiences among TechMates. Lastly, selection bias may have been introduced due to recruitment methods of participants and volunteers, as well as the absence of screening measures for social isolation, loneliness, and prior technology knowledge or use.

## Conclusion

In light of physical distancing resulting from the COVID-19 pandemic, older adults may benefit from technology-supported social interactions. As community-based organizations and researchers seek to address social isolation and loneliness in homebound older adults, they must consider organization-level implementation facilitators and barriers to develop sustainable and effective programs. The facilitators, barriers, and lessons learned identified in Talking Tech can inform development and implementation of ICT training programs by other community-based organizations and researchers to support homebound older adults both during and beyond the pandemic.

## Data Availability Statement

The raw data supporting the conclusions of this article will be made available by the authors, without undue reservation.

## Author Contributions

JB, LF, EG, and KT contributed to conception and design of the intervention program. JB, EG, and KT contributed to conception and design of the study. FJ acquired the data and prepared the manuscript. JB, ND, EG, and FJ analyzed and interpreted study data. All authors contributed to manuscript revision, read, and approved the submitted version.

## Funding

This work was supported by Meals on Wheels America and the Aetna Foundation. Meals on Wheels America was involved in conception and design of the intervention, reviewed the manuscript, and approved the submitted version. MOWA was not involved in any aspect of study design, acquisition of subjects and/or data, or analysis and interpretation of data. The Aetna Foundation was not involved in any aspect of study concept and design, acquisition of subjects and/or data, analysis and interpretation of data, or preparation of manuscript.

## Conflict of Interest

The authors declare that the research was conducted in the absence of any commercial or financial relationships that could be construed as a potential conflict of interest.

## Publisher's Note

All claims expressed in this article are solely those of the authors and do not necessarily represent those of their affiliated organizations, or those of the publisher, the editors and the reviewers. Any product that may be evaluated in this article, or claim that may be made by its manufacturer, is not guaranteed or endorsed by the publisher.

## Footnotes

^1^ https://www.qsrinternational.com/nvivo/home
