# Supplementary material for: A Technology Training Program to Alleviate Social Isolation and Loneliness Among Homebound Older Adults: A Community Case Study
Source: Front Public Health. 2021 Nov 18;9:750609. doi: 10.3389/fpubh.2021.750609 (PMC8637200; doi:10.3389/fpubh.2021.750609)
Supplement: Supplementary file 1 [file Table_1.DOCX]

**Supplementary File 1. TechMate Report Template**

**Meals on Wheels Rhode Island**

**Tech to Connect**

**Tech Mate Report**

Please take a few moments to complete the following evaluation after each visit/call with your client participant. Detail is helpful, but not all questions will be applicable for each visit/call. Thank you!

Volunteer Tech Mate Name: ___________________________________________

Participant Name: ___________________________________________________

Date of Contact: _____________________________________________________

1. Was this a visit or call? (Please circle one)

2. How long did your visit/call last?

4. What did you and your participant talk about? What questions did they have for you?

5. Compared to last time, does your participant seem to be getting more comfortable with the technology?

6. Compared to last time, does your participant seem to be more interested in using technology or connecting to Well Connected?

7. Did you have any challenges this week? Do you have any ideas for how we can improve this program?
